# The effect of time-of-day on short-term repetitive maximal performance, cognitive ability and psychological variables in adolescents

**DOI:** 10.1371/journal.pone.0300897

**Published:** 2025-03-26

**Authors:** Rawdha Hasni, Ibrahim Ouergui, Achraf Ammar, Khaled Trabelsi, Luca Paolo Ardigò, Hamdi Chtourou

**Affiliations:** 1 High Institute of Sport and Physical Education, University of Sfax, Sfax, Tunisia; 2 Research Laboratory “Education, Motricité, Sport et Santé” (EM2S) LR19JS01, High Institute of Sport and Physical Education of Sfax, University of Sfax, Sfax, Tunisia; 3 High Institute of Sport and Physical Education of Kef, University of Jendouba, El Kef, Tunisia; 4 Research Unit, Sports Science, Health and Movement, University of Jendouba, El Kef, Tunisia; 5 Department of Training and Movement Science, Institute of Sport Science, Johannes Gutenberg-University Mainz, Mainz, Germany; 6 Interdisciplinary Laboratory in Neurosciences, Physiology and Psychology: Physical Activity, Health and Learning (LINP2), UFR STAPS (Faculty of Sport Sciences), UPL, Paris Nanterre University, Nanterre, France; 7 Research Laboratory, Molecular Bases of Human Pathology, LR19ES13, Faculty of Medicine, University of Sfax, Sfax, Tunisia; 8 Department of Teacher Education, NLA University College, Oslo, Norway; 9 Physical Activity, Sport, and Health, UR18JS01, National Observatory of Sport, Tunis, Tunisia; Arba Minch University, ETHIOPIA

## Abstract

Previous studies investigating the diurnal variation of physiological and psychological variables during exercise have yielded conflicting results. The present investigation was designed to assess the impact of time-of-day on short-term repetitive maximal performance [*i.e.,* single and two consecutive bouts of 5m shuttle run test (5mSRT), long jump test] as well as cognitive ability and psychological variables [*i.e.,* mood states (POMS) and Hooper questionnaire] in male and female adolescents. In a randomized study design, 21 healthy adolescents (12 females and 9 males; age: 15.9±1.04 years) performed at 08h00 and 16h00 two consecutive 5mSRT (with 20 min of rest interval in-between), to assess the greatest distance (GD), the total distance (TD), the average distance (AD) and the fatigue index (FI), and the long jump test (LJT). Perceived exertion (RPE) was recorded immediately after each 5mSRT. The POMS and Hooper questionnaires and the digit-cancellation test (*i.e.,* attention) were realized during each session. The results showed that TD and AD were greater in the morning than in the afternoon during the 1^st^ (p = 0.048 and 0.048, respectively) and the 2^nd^ (p = 0.0016 and 0.0017, respectively) 5mSRT; while GD was time-of-day independent (p = 0.16). For the FI, results demonstrated a significant time-of-day effect with highest values recorded in the afternoon compared to the morning during the 2^nd^ 5mSRT (p = 0.017). In contrast, attention scores and the long jump test performance recorded before and after each 5mSRT were time-of-day independent for all measures (p > 0.05). Likewise, POMS parameters [anxiety (p = 0.15), depression (p = 0.71), anger (p = 0.23), fatigue (p = 0.07), confusion (p = 0.58), vigor (p = 0.16), TMD (p = 0.06) and interpersonal relationships (p = 0.19)], RPE [1^st^ (p = 0.22) and 2^nd^ (p = 0.43) 5mSRT] and delayed onset muscle soreness (p = 0.34) were time-of-day independent; while, fatigue (p = 0.03), sleep (p = 0.01) and stress (p = 0.027) estimated by the Hooper questionnaire were higher in the afternoon compared to the morning. In conclusion, morning is more effective than afternoon session for improving short-term repetitive maximal performance and reducing fatigue during the 5mSRT for adolescents. However, regarding psychological parameters and cognitive function and contrarily to previous researches, there is no time-of-day effects.

## Introduction

The human body operates on a circadian rhythm regulated by the suprachiasmatic nucleus, governing various physiological and biochemical functions such as the sleep-wake cycle that are associated with optimal performances [1]. According to the literature, many physical parameters, such as repeated sprint ability, muscle strength, anaerobic power, multiple jumps, and fatigue are time dependent [1,2]. In this context, Belkhir et al. [3], for physical performance, reported that the best and the total distance covered during the 5m shuttle run test (i.e., 6×30-s with 35-s recovery) were significantly better in the afternoon compared to the morning. Likewise, Testu and Bréchon [4] reported a significant time-of-day effects on cognitive performance, with higher values in the afternoon compared to the morning.

Despite extensive research on the diurnal variation of physical performance in adults, adolescents have been less studied in this context [5]. Given the significant physiological and psychological changes that occur during adolescence, it is plausible that these factors could influence daily variations in physical and cognitive performance within this age group [6]. In this context, Goldstein et al. [7] highlighted the impact of time-of-day on adolescents’ cognitive performance and behavior, emphasizing variability influenced by individuals’ circadian preferences. Ouergui et al. [8] indicated that both dynamic and isometric judo chin-up tests show time-of-day variations, with adolescents performing better in the afternoon than in the morning. These diurnal variations in performance can be attributed to various factors, including perceptual and psychological responses such as decreased perception of effort, along with improvements in cognitive performance (*i.e.,* reaction time) and vigor [8].

To explain these diurnal changes in physical and cognitive performances, previous studies have investigated the perceptual and psychological responses to exercises at different time-of-day [9,10]. In this context, it has been reported that the scores of fatigue estimated by the Hooper questionnaire and the perceived exertion (RPE) were higher in the afternoon compared to the morning [11]. Also, Hill and Chtourou [9] revealed that changes in performance from morning to afternoon were correlated with fluctuations in vigor and fatigue among University students. Moreover, Chtourou et al. [10] reported that, during repeated high-intensity efforts, the independence of most psychological markers, such as mood states, was associated with a lack of significant diurnal variations in performance and fatigue.

Adolescence, in particular, introduces physiological and psychological changes that affect daily performance fluctuations [6]. These changes are linked to a longer circadian period, delayed sleep onset, and reduced sleep pressure during waking hours, which allow adolescents to stay awake longer than adults [12]. Furthermore, the interplay between brain mechanisms, circadian rhythms, and puberty, including variations in melatonin and gonadotropin levels [13], likely contributes to performance fluctuations, underscoring the importance of identifying optimal training times [14].

According to the authors’ current knowledge, no study has investigated the diurnal variation of these aspects aforementioned in adolescents. Therefore, the present study aimed to investigate the diurnal fluctuation of short-term repetitive maximal performance as well as mood, fatigue, stress, sleep, muscle soreness and constant attention in adolescents. We hypothesized that physical and cognitive performances are better in the afternoon and that these diurnal variations are related to perceptual and psychological responses.

## Materials and methods

### Participants

Twenty-one adolescents (12 females and 9 males; mean ± SD: age: 15.9 ± 1.04 years; body mass: 59.87 ± 9.04 kg; height: 1.68 ± 0.07 m; BMI: 20.87±1.99 kg • m^-^²) volunteered to participate in the present study. They were participating regularly in physical education classes for 2 sessions/week, with each one lasting 2h. Participants and their parents were informed about the protocol and signed a written informed consent before their participation. The present study was conducted in accordance to the Declaration of Helsinki and the protocol was fully approved by the University of Sfax research ethics committee (CPP SUD n° 0224/2020). The current study was carried out in Tunisia in 2021.

### Experimental design

One week before the beginning of the experimentation, participants were familiarized with the equipment and the experimental protocol. The measurement of body mass and height was also done. After that, participants randomly assigned into two test sessions (Morning: 08h00 and afternoon: 16h00) with at least 48h of rest in-between. During each session, subjects started by answering the profile of mood states (POMS) questionnaire using the French version by Cayrou et al. [15] and performing the digit cancellation test [16].

**Fig 1:**
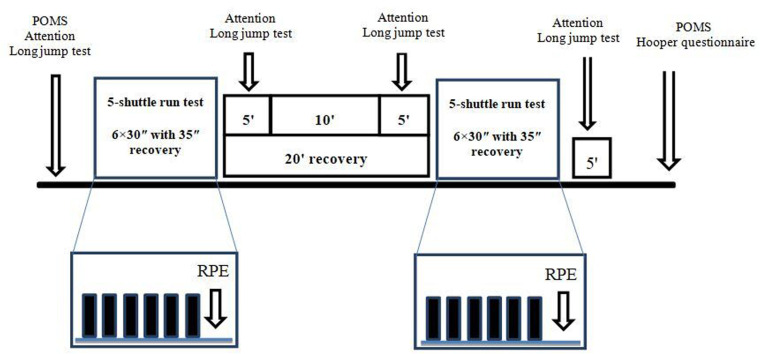
Experimental design. POMS = profile of mood states questionnaire, RPE = rate of perceived exertion.

After that, they completed 10 min of standardized warm-up (5 min of jogging at a moderate intensity followed by 5 min of dynamic stretching and four progressive accelerations that covered 100 m). Then, participants performed the long jump test (LJT) followed by two 5m shuttle run test (5mSRT) with a 20 min of rest in between. At the end of each 5mSRT, participants were asked immediately to rate their perceived exertion (RPE). Additionally, participants performed the long jump test and answered the digit cancellation test at 5 min after the 1^st^ 5mSRT and at 5 min before and after the 2^nd^ 5mSRT. Participant answered the POMS questionnaire a second time and finally the Hooper questionnaire in order to provide a subjective estimation of the prior night sleep as well as fatigue, stress and delayed onset muscle soreness (DOMS) scores [17]. The experimental design is presented in Fig 1.

### Measures

#### Profile of mood state questionnaire.

The POMS questionnaire is a series of 65- adjectival items designed to assess seven mood states (*i.e.,* five negative moods: anxiety, depression, anger, fatigue, confusion; one positive mood: vigor and interpersonal relationship). Responses to each item range from “0” (Not at all) to “4” (Extremely), with higher scores indicating more negative mood. The total mood disturbance (TMD) score consists of adding the answers given to the items corresponding to each mood state except interpersonal relationship [TMD = (Tension + Depression + Anger + Fatigue + Confusion) – Vigor] [15].

#### Hooper questionnaire.

The Hooper questionnaire was designed to provide a subjective estimation of the prior night sleep as well as fatigue, stress and DOMS scores [17] The four subjective evaluations were measured using a scale ranging from 1 (indicate very, very good for sleep; very, very low of fatigue, stress and DOMS) to 7 (represented very, very bad for sleep quality, very, very high for fatigue, stress and DOMS) [18].

#### Digit cancellation.

The digit cancellation (*i.e.,* paper-pencil test) is a psychometric task which evaluates information processing speed, the ability to focus attention and executive functioning. It consists of 4 different shapes (A, B, C, and D) so that the subject cannot memorize the contents of the shapes already made. This content consists of a number composed of 2 to 5 digits. Participant must check off as many as possible 3-digit numbers within one minute [16]. The number of correct answers was retained for the statistical analyzes.

#### Rating of perceived exertion scale.

This RPE was assessed using the category-ratio scale of Borg (Borg CR10 scales). This RPE scale ranging from “1” to “10”, with corresponding verbal expressions, that gradually increase with the intensity of perceived sensation (1 = Very, Very Easy; 2 = Very Easy; 3 = Easy, 4 = Just Feeling a Strain; 5 = Starting to Get Hard; 6 = Getting Quite Hard; 7 = Hard; 8 = Very Hard; 9 = Very, Very Hard; 10 = So Hard I am Going to Stop) [19].

#### The long jump test.

The long jump test was carried out in the field to determine the performance in meter and it reflects the explosive strength of the lower limbs. The participant started behind the call line, feet slightly apart, before the jump he bends the knees with his arms extended backward with elbows bent. He jumps, rushing forward as far as possible, by exercising a phase of flight during which he brings the legs back to the front and straightens the knees and simultaneously swinging the arms. The distance jumped was measured using a tape [20]. The long jump test has been well-established in previous literature for its reliability and validity in assessing lower body power, particularly in adolescent populations. A previous study has demonstrated high test-retest reliability (r = 0.96 and 0.90) and concurrent validity both in male and females subject [20].

#### 5m shuttle run test.

The 5mSRT was utilized to assess participant’ agility and speed. It has been widely validated and demonstrated to be a reliable measure of both total distance (r = 0.98) and peak distance (r = 0.86) [21]. The 5mSRT is a series of six repetitions of 30-s maximal shuttle sprints of increasing distances: 5 m, 10 m, 15 m, 20 m and 25 m. The covered distance during each repetition was registered and the total distance (TD) as well as the greatest distance (GD), the average distance (AD) and the fatigue index (FI) were calculated according to Boddington et al. [21]:

- Total distance (TD) (m) = sum of distances covered during the 6 × 30-s shuttles
- The average distance (AD) (m) = TD/6
- Greatest distance (GD) (m) = highest distance covered during one of the 6 × 30-s shuttles
- Fatigue index (FI) (%) $=(\frac{\left[\left(\text{shuttle }1+\text{shuttle }2\right)-\left(\text{shuttle }5+\text{shuttle }6\right)\right]}{\left(\text{shuttle }1+\text{shuttle }2\right)}$ × 100

### Statistical analyses

The statistical analysis was performed using STATISTICA Software (StatSoft, France, version 10). Data were presented as mean and standard deviation and Median and Interquartile range values were reported for non-normal distribution data. The Shapiro-Wilk test was used to check and confirm the normality of data sets.

For normally distributed data (*i.e.,* TD and the AD recorded during the 5mSRT, confusion, anger, fatigue, interpersonal relationship, vigor and TMD) a two-way- ANOVA (time-of-day × exercise) with repeated measures was performed. As well, a one-way ANOVA was performed for fatigue and DOMS. Post hoc comparisons were made using the Bonferroni test.

For non-normally distributed data (*i.e.,* GD, FI and RPE scores recorded during the 5mSRT, anxiety and depression, sleep and stress) the Friedman non-parametric test was applied. Pairwise comparisons were conducted using a Wilcoxon test.

Effect size for the normally distributed variables were calculated as partial eta-squared (η_p_^2^) to estimate the meaningfulness of significant findings. Partial eta squared (η_p_^2^) effect size values were reported and classified as 0.01 = small, 0.06 = medium, 0.14 = large [22]. For the non-normally distributed variables, effect size was estimated by the Kendall’s W coefficient of concordance. The level of statistical significance was set at p ≤ 0.05.

## Results

### 5-m Shuttle run test

#### Total distance.

**Fig 2:**
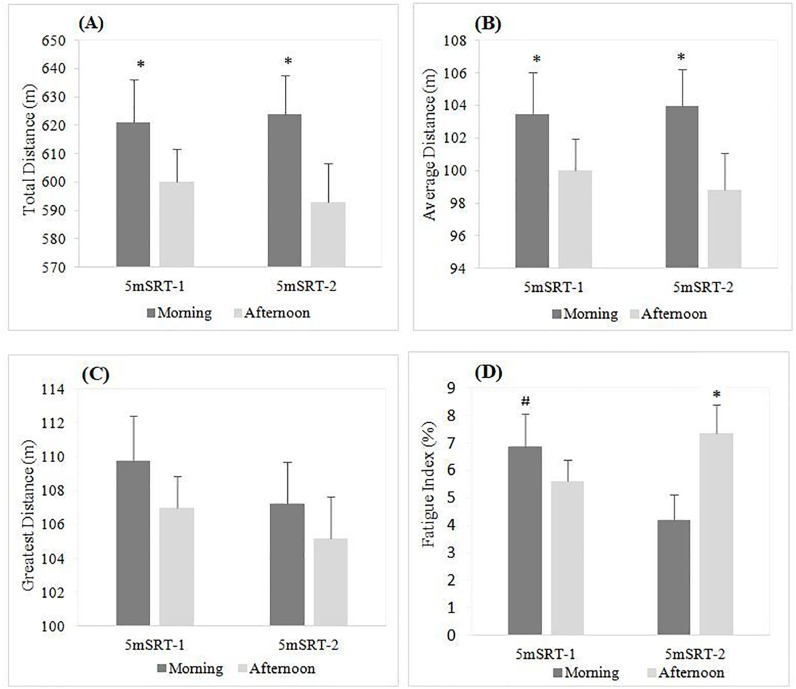
Morning and afternoon total, average and greatest distance and fatigue index. 5mSRT-1 = 1^st^ 5m shuttle run test, 5mSRT-2 = 2^nd^ 5m shuttle run test.

Statistical analysis revealed a significant effect of time-of-day (F₁, _2_₀ = 10.37, p = 0.004, η_p_^2^ = 0.34). The post hoc analysis showed that TD was significantly higher in the morning compared to the afternoon during the 1^st^ (p = 0.048) and the 2^nd^ (p = 0.0016) 5mSRT. However, no significant differences between the two exercises were registered (F₁, _2_₀ = 0.20, p = 0.65, η_p_^2^ = 0.01). Likewise, there was no exercise × time-of-day interaction effect (F₁, _2_₀ = 1.056, p = 0.31, η_p_^2^ = 0.05) (Fig 2).

#### Average distance.

There was a significant main effect of time-of-day (F₁, _2_₀ = 10.334, p = 0.004, η_p_^2^ = 0.34). The post hoc test showed that AD was significantly higher in the morning compared to the afternoon during the 1^st^ (p = 0.048) and the 2^nd^ (p = 0.0017) 5mSRT. However, there was no significant main effect of exercise (F₁, _2_₀ = 0.2, p = 0.65, η_p_^2^ = 0.009). Additionally, there was no interaction effect between exercise and Time-of-day (F₁, _2_₀_)_ = 1.05, p = 0.31, η_p_^2^ = 0.049) (Fig 2).

#### Greatest distance.

The Friedman test revealed no significant effect of time-of-day and exercise (Chi^2^ = 5.11, p = 0.16, Kendall’s W = 0.081) (Fig 2).

#### Fatigue index.

Friedman test revealed a significant effect (Chi^2^ = 9.05, p = 0.02, Kendall’s W = 0.14). Pairwise comparisons showed that FI was significantly higher in the afternoon compared to the morning during the 2^nd^ 5mSRT (p = 0.017). In addition, significant differences between the two exercises in the morning with highest values were registered during the 1^st^ 5mSRT (p = 0.018) (Fig 2).

### Rating of perceived exertion

**Fig 3:**
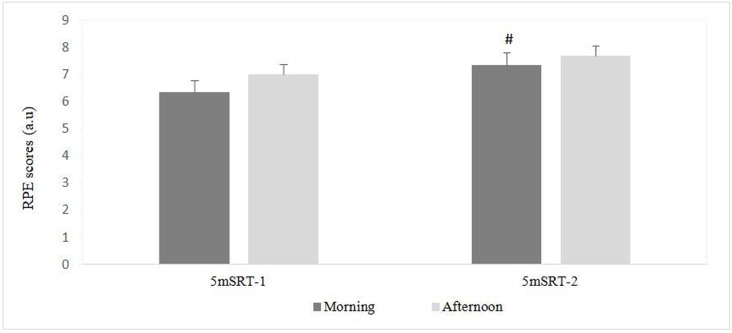
Morning and afternoon rate of perceived exertion. RPE = rate of perceived exertion, 5mSRT-1 = 1^st^ 5m shuttle run test, 5mSRT-2 = 2^nd^ 5m shuttle run test.

A Friedman test conducted on RPE scores reported a significant effect (Chi^2^ = 8.07, p = 0.04, Kendall’s W = 0.12). Pairwise comparisons revealed no significant differences between the two times of day during the 1^st^ (p = 0.22) and the 2^nd^ (p = 0.43) 5mSRT. However, there were significant differences between the two exercises in the morning with the highest values recorded during the 2^nd^ 5mSRT (p = 0.015) (Fig 3).

### Long jump test

**Fig 4:**
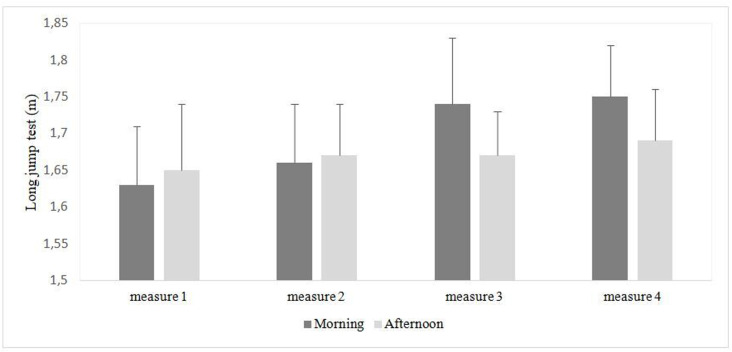
Morning and afternoon long jump measures.

A significant effect of measure was recorded (F₁, _2_₀ = 2.89, p = 0.04, η_p_^2^ = 0.12). The Bonferroni test revealed significant differences between the 1^st^ measure and the 3^rd^ measure in the morning with highest values registered during the 3^rd^ measure (p = 0.037). Additionally, the Bonferroni test showed significant differences between the 1^st^ measure and the 4^th^ measure in the morning with highest values registered during the 4^th^ measure (p = 0.009). However, there was no significant main effect of time-of-day (F_2_, 60 = 1.31, p = 0.26, η_p_^2^ = 0.06). Likewise, there was no time-of-day × measure interaction (F_3_, 60 = 2.38, p = 0.07, η_p_^2^ = 0.1) (Fig 4).

### Attention scores

**Fig 5:**
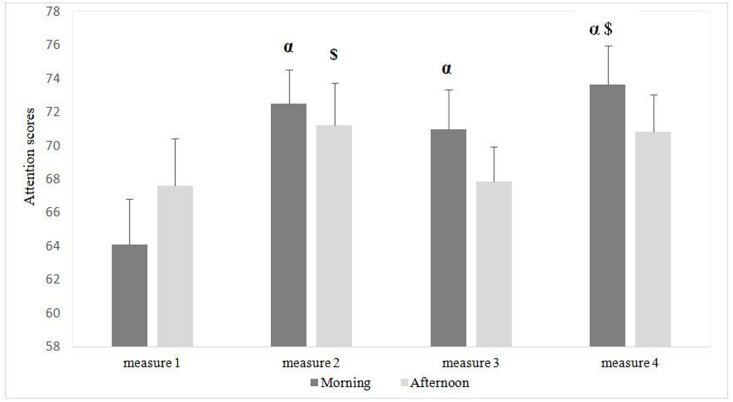
Morning and afternoon long attention scores.

Statistical analysis reported no significant main effect of time-of-day (F₁, _3_₀ = 0.29, p = 0.59, η_p_^2^ = 0.014). However, there was a significant main effect of measure (F_3_, 60 = 12.009, p = 0.000003, η_p_^2^ = 0.37). The post hoc analysis showed significant differences between the attention scores recorded during the 1^st^ and the 2^nd^ measure in the morning with highest values during the 2^nd^ one (p = 0.0005). Also, there were significant differences between the attention scores recorded during the 1^st^ measure and the 3^rd^ measure in the morning with highest values during the 3^rd^ measure (p = 0.009) as well as between the 1^st^ measure and the 4^th^ measure in the morning with highest values during the 4^th^ measure (p = 0.00005) (Fig 5).

Furthermore, statistical analysis revealed a significant time-of-day × measure interaction (F_3_, 60 = 2.87, p = 0.04, η_p_^2^ = 0.12). The Bonferroni test revealed significant differences between the 1^st^ measure in the morning and the 2^nd^ measure in the afternoon with highest values registered in the afternoon (p = 0.005). Likewise, there were significant differences between the 1^st^ measure in the afternoon and the 4^th^ measure in the morning with highest values during the 4^th^ one (p = 0.043) and finally between the 1^st^ measure in the morning and the 4^th^ measure in the afternoon with highest values during the 4^th^ one (p = 0.012).

### Profile of mood states questionnaire

There were no significant differences between the two times of day for anger, fatigue, as well as confusion, vigor, TMD and interpersonal relationships (Table 1). Similarly, no significant differences between the beginning and the end of session were registered for anger, confusion and TMD (Table 1). Also, no significant differences between the beginning and the end of session in the morning and afternoon for fatigue as well as for vigor and interpersonal relationships were reported (p > 0.05). Statistical analysis revealed no significant differences for anger, confusion, fatigue, vigor, interpersonal relationships and TMD according to the session and the time-of-day (Table 1).

**Table 1:** Anger, confusion, fatigue, vigor, anxiety, depression, interpersonal relationship and total mood disturbances (TMD) scores assessed by the profile of mood states questionnaire during each session in the morning and the afternoon.

	Beginning of the session		End of the session		ANOVA		
	**Morning**	**Afternoon**	**Morning**	**Afternoon**	**Time-of-day**	**Measure**	**Interaction**
Anger (a.u.)	5.71 ± 6.39	6.43 ± 6.14	5.76 ± 5.98	6.24 ± 3.92	F_1,20_ = 1.5, p = 0.23, η_p_^2^ = 0.07	F_1,20_ = 0.018, p = 0.89, η_p_^2^ = 0.0009	F_1,20_ = 0.078, p = 0.78, η_p_^2^ = 0.003
Confusion (a.u.)	5.38 ± 4.14	6.10 ± 3.92	5.24 ± 3.48	5.29 ± 2.70	F_1,20_ = 0.31, p = 0.58, η_p_^2^ = 0.01	F_1,20_ = 0.68, p = 0.41, η_p_^2^ = 0.03	F_1,20_ = 0.53, p = 0.47, η_p_^2^ = 0.026
Fatigue (a.u.)	3.90 ± 4.68	5.48 ± 5.44	5.43 ± 4.30	6.71 ± 5.09	F_1,20_ =3.48, p = 0.07, η_p_^2^ = 0.14	F_1,20_ = 6.90, p = 0.01, η_p_^2^ = 0.25	F_1,20_ = 0.05, p = 0.81, η_p_^2^ = 0.002
Vigor (a.u.)	24.00 ± 4.60	21.90 ± 5.4	21.14 ± 6.38	20.86 ± 5.8	F_1,20_ = 2.08, p = 0.16, η_p_^2^ = 0.09	F_1,20_ = 4.58, p = 0.04, η_p_^2^ = 0.18	F_1,20_ = 1.57, p = 0.22, η_p_^2^ = 0.07
Interpersonal relationship (a.u.)	18.81 ± 4.66	20.10 ± 5.3	16.95 ± 5.38	17.71 ± 5.1	F_1,20_ = 1.82, p = 0.19, η_p_^2^ = 0.08	F_1,20_ = 10.68, p = 0.003, η_p_^2^ = 0.34	F_1,20_ = 0.16, p =0.68, η_p_^2^ = 0.008
Total mood disturbances score (a.u.)	-0.71 ± 21.98	5.52 ± 26.8	3.00 ± 19.15	5.90 ± 13.2	F_1,20_ = 3.69, p = 0.06, η_p_^2^ = 0.15	F_1,20_ = 0.59, p = 0.45, η_p_^2^ = 0.02	F_1,20_ = 0.62, p = 0.43, η_p_^2^ = 0.03
					**Friedman test**		
Anxiety (a.u.)	3 (1; 7)	5 (3; 7)	4 (2; 6)	4 (4; 7)	Chi^2^ = 5.20, p = 0.15, Kendall’s W = 0.082		
Depression (a.u.)	1 (0; 3)	1 (0; 4)	1 (0; 3)	1 (0; 3)	Chi^2^ = 1.34, p = 0.71, Kendall’s W = 0.021		

Note: values are presented as means ± SD for anxiety and depression, values are presented as Median (Quartile1; quartile 3); a.u arbitrary units.

Concerning anxiety and depression, Friedman test showed no significant differences between the two times and between the beginning and the end of session (Table 1).

### Hooper questionnaire

Fatigue, DOMS, sleep and stress scores are presented in Table 2.

**Table 2:** Fatigue, delayed onset muscle soreness (DOMS), stress and sleep recorded in the morning and the afternoon by the Hooper questionnaire.

	Morning	Afternoon	ANOVA
Fatigue (a.u.)	3.05 ± 0.86	3.62 ± 1.12^*^	F_1,20_ = 5.45, p = 0.03, η_p_^2^ = 0.21
DOMS (a.u.)	2.86 ± 1.31	3.24 ± 1.37	F_1,20_ = 0.93, p = 0.34, η_p_^2^ = 0.04
			**Friedman test**
Stress (a.u.)	2 (2; 3)	3 (3; 4)^*^	Chi^2^ = 6.25, p = 0.01, Kendall’s W = 0.29
Sleep (a.u.)	2 (1; 3)	3 (3; 3)^*^	Chi^2^ = 7.11, p = 0.007, Kendall’s W = 0.33

Note: values are presented as mean ± SD for stress and sleep, values are expressed as median (Quartile1; quartile 3);

^*^Significant difference for comparison of the time-of-day; a.u arbitrary units*.*

#### Fatigue.

ANOVA test reported a significant effect of time-of-day (Table 2). The post hoc analysis showed that fatigue was significantly higher in the afternoon compared to the morning (p = 0.03).

#### DOMS.

ANOVA test revealed no significant effect of time-of-day (Table 2).

#### Sleep.

Friedman test revealed a significant main effect of time-of-day (Table 2). The statistical analysis showed that sleep was significantly higher in the afternoon compared to the morning (p = 0.01).

#### Stress.

Friedman test revealed a significant main effect of time-of-day (Table 2). The statistical analysis showed that the stress was significantly higher in the afternoon compared to the morning (p = 0.027).

Raw data are shown in S1 Table.

## Discussion

The main findings of the current study were: (*i*) TD and AD recorded during the 5mSRT followed a diurnal variation with values significantly higher in the morning compared to the afternoon during the 1^st^ and 2^nd^ 5mSRT, while GD was time-of-day independent, (*ii*) FI was significantly higher in the afternoon compared to the morning during the 2^nd^ 5mSRT, (*iii*) attention, long jump test as well as mood state, RPE and DOMS were time-of-day independent and (*iv*) fatigue, sleep and stress estimated by the Hooper questionnaire were higher in the afternoon compared to the morning.

In contradiction with the present study’ findings concerning TD and AD during the 5mSRT, Belkhir et al. [3] and Souissi et al. [23] reported higher TD in the afternoon. Additionally, Pullinger et al. [24] reported better repeated sprint performance on a non-motorized treadmill in the afternoon compared to the morning with an average time-of-day difference of ~8% in distance covered, average power, and average velocity. However, Chtourou et al. [10] showed that performance during a repetitive sprint exercise was not significantly different between the morning and afternoon.

The extent of daily performance variation is influenced by various factors, including fitness level [25], chronotype [26] and social activities [27]. Considering the participants’ fitness levels, Atkinson et al. [25] reported a greater morning-afternoon difference in performance in physically active compared to inactive participants. However, this depends on the time-of-day of training [2] and the chronotype [26]. Indeed, adolescents shift from morningness to eveningness between 12 and 14 years [28]. However, early school schedules can disrupt natural sleep patterns in children and adolescents [29] and, therefore, this shift in chronotype. In the present study, participants reported better perceived sleep quality measured by the Hooper questionnaire in the morning. All these factors could, in part, explain the higher performance observed in the morning.

For the GD during the 5mSRT, the present study’ results showed that performance was not affected by the time-of-day in agreement with the previous study of Chtourou et al. [10] and Giacomoni et al. [30] who observed no significant diurnal variation in performance during repeated sprint tests. This non significant time-of-day effect could be attributed to the pre-exercise warm-up. Indeed, Souissi et al. [31] reported that the amplitude of the diurnal variation in short-term maximal performance was reduced after a 10 min warm-up (the duration used in the present study). Also, this warm-up could explain the absence of a diurnal variation of the long jump performance.

Indeed, it has been suggested that higher core temperature in the afternoon enhance these peripheral mechanisms of muscular contraction, thus increasing short-term maximal performance [32].

Furthermore, in consistency with the results of previous studies [23,33,34], FI recorded during the present study was higher in the afternoon compared to the morning during the 2^nd^ 5mSRT which suggests that athletes are more exhausted in the afternoon when performing maximum and repeated efforts. Likewise, subjective fatigue recorded by the Hooper questionnaire was higher in the afternoon compared to the morning. The improved physical performance during the 5mSRT and reduced fatigue observed in the morning may be partially attributed to the better sleep quality perception at this time-of-day. Also, stress scores measured by the Hooper questionnaire were significantly higher in the afternoon compared to the morning.

However, the present study did not report a significant difference between the morning and afternoon for fatigue scores recorded by the POMS questionnaire, RPE scores and muscle soreness scores estimated by the Hooper questionnaire. Also, mood states recorded by POMS questionnaire subscales (*i.e.,* tension, depression, anger, confusion, vigor, interpersonal relationship and total mood score) were not affected by the time-of-day. Thus, all these parameters are not related to changes in performances.

Regarding attention, the present study displayed no significant difference between morning and afternoon. This result is not in agreement with those previously reported by Jarraya et al. [35], which showed that constant attention in Tunisian children was time-of-day dependent, with better values recorded at 09h00. Similarly, Jarraya et al. [36] demonstrated that cognitive performance (*i.e.,* selective and constant attention) in handball goalkeepers were time-of-day dependent, with higher performance being recorded in the morning. These differences may be due to the days chosen for testing, as Jarraya et al. [35] noted that midweek days like Thursdays often align with peak school performance. Additionally, individual circadian preferences, with morning types are better in the morning and evening types performing better later in the day, significantly influence performance. Previous studies, such as those by Schmidt et al. [37] and Goldstein et al. [7] have shown that cognitive performance and behavioral issues vary according to circadian preferences. Finally, we acknowledge some limitations in the present study. Specifically, we did not control for the menstrual cycle of adolescent girls. The menstrual cycle can affect body temperature, hormone levels, mood, and sleep patterns, potentially influencing daily performance variations. Future research should include menstrual cycle tracking to more accurately understand these factors and their impact on performance, especially in adolescents [5].

Another limitation of the study was that we analyzed the response to exercise conducted only at two times of the day (*i.e.,* 08h00 and 16h00). Therefore, future studies should involve conducting testing and measurements more frequently throughout the day, such as every 2 h, *e.g.,* at 07h00, 09h00, 11h00, 13h00, 15h00 h, to allow better understanding of the whole diurnal variation.

The lack of motivation assessment was a significant limitation of the study. Circadian rhythm affects motivation, and high levels of motivation are associated with better cognitive performance especially for this age category. Thus, the inclusion of subjective motivation measurement in future research could assist in the interpretation of data.

One additional limitation worth mentioning is the lack of assessment of participants chronotype, which affects circadian rhythms and performance. Chronotype, influenced by age and sex, typically shows a morning preference as individuals age and varies between genders [26]. This parameter could vary also according the school schedules (*i.e.,* vacation, non-vacation). Future research should include chronotype assessment to better understand its impact on physical and mental performance among adolescent athletes [38]. Furthermore, the lack of body temperature measurement was a significant limitation of the study, as it prevented us from assessing whether it changes according to the time-of-day and restricted our understanding of how body temperature fluctuations may have influenced our results. Indeed, it has been reported that physical performance can increase by approximately 5% with a 1°C rise in muscle temperature, particularly due to increased enzymes activity such as phosphofructokinase and lactate dehydrogenase [39].

## Conclusions

The results of the present study highlighted a diurnal variation of physical performance measured during two 5mSRT tests with highest values of TD and AD in the morning; while GD was time-of-day independent. Furthermore, FI recorded during the 5mSRT and the subjective fatigue measured by the Hooper questionnaire were time-of-day dependent with higher values in the afternoon. Additionally, the sleep scores estimated by the Hooper questionnaire was better in the morning. In general, better physical performance and lower fatigue in the morning can be linked to a good sleep quality observed in the morning. Additionally, higher stress score recorded in the afternoon could explain the impaired performance in the afternoon as well as the fatigue increases. Therefore, based on our findings of enhanced physical performance, specifically in terms of total distance and average distance, during morning assessments, we recommend scheduling training sessions involving repeated sprints accordingly. However, the present study reported that attention, long jump performance, mood states, perceived exertion and muscle soreness were time-of-day dependent.

## Supporting information

S1 Table Full raw data. (XLSX)
